# A molecular survey, whole genome sequencing and phylogenetic analysis of astroviruses from roe deer

**DOI:** 10.1186/s12917-020-02289-4

**Published:** 2020-02-21

**Authors:** Urska Jamnikar-Ciglenecki, Vita Civnik, Andrej Kirbis, Urska Kuhar

**Affiliations:** 1 Institute of Food safety, Feed and Environment, University of Ljubljana, Veterinary faculty, Gerbičeva 60, 1000 Ljubljana, Slovenia; 2 Institute of Microbiology and Parasitology, University of Ljubljana, Veterinary faculty, Gerbičeva 60, 1000 Ljubljana, Slovenia

**Keywords:** Astrovirus, Mamastrovirus, Roe deer, Phylogenetic analysis, Wildlife, Next generation sequencing, Taxonomic classification

## Abstract

**Background:**

Although astroviruses (AstV) have been detected in a variety of host species, there are only limited records of their occurrence in deer. One of the most important game species in Europe, due to its meat and antlers, is roe deer. Infected game animals can pose a threat to the health of other animals and of humans, so more attention needs to be focused on understanding the diversity of viruses in wildlife. The complete genome and organization of the roe deer AstV genome have not so far been described.

**Results:**

In our study, 111 game animals were screened for the presence of AstV. While no AstVs were detected in red deer, wild boar, chamois and mouflon, AstV RNA was present in three samples of roe deer. They were further subjected to whole genome sequencing with next generation sequencing. In this study, two AstV genomes were assembled; one in sample D5–14 and one in sample D12–14, while, in sample D45–14, no AstV sequences were identified. The complete coding sequences of the AstV SLO/D5–14 strain genome and of the almost complete genome of the AstV SLO/D12–14 strain were determined. They showed a typical Mamastrovirus organization. Phylogenetic analyses and amino acid pairwise distance analysis revealed that Slovenian roe deer AstV strains are closely related to each other and, also, related to other deer, bovine, water buffalo, yak, Sichuan takin, dromedary, porcine and porcupine AstV strains - thus forming a highly supported group of currently unassigned sequences.

**Conclusions:**

Our findings suggest the existence of a new Mamastrovirus genogroup might be constituted while this aforementioned group is distantly related to Mamastrovirus genogroups I and II. In this study, additional data supporting a novel taxonomic classification are presented.

## Background

Astroviruses (AstV) are small, round, non-enveloped viruses (28–30 nm in diameter), often with a distinct five- or six-pointed star-like appearance under the electron microscope (EM) [1, 2]. They have a single stranded, positive-sense RNA genome, 6.4–7.9 kb in length, that forms three open reading frames (ORF), ORF1a, ORF1b and ORF2. ORF1a and ORF1b encode non-structural polyproteins (nsp1a and nsp1ab) that are presumed to be involved in RNA transcription and replication, while ORF2 encodes the capsid (CA) proteins [3–5]. The length of all these ORFs varies in different AstVs strains, the largest variations being observed in ORF1a due to the presence of insertions and deletions near the 3′ end [6]. The organization of the genome begins with a short (11 to 85 nt) 5′ untranslated region (UTR) that precedes ORF1a and which encodes nsp1a with a protease motif. The Nsp1a characteristic features are also the presence of transmembrane (TM) domains, coiled coil (CC) structures, a putative viral genome-linked protein (VPg) coding region and a potential nuclear localization signal (NLS) [6]. Nsp1ab is translated from ORF1a and ORF1b through a ribosomal frameshift mechanism (5′-AAAAAAC-3′) [6]. ORF1a and ORF1b overlap by 10 to 148 nucleotids (nt) in the genomes of mammalian AstV. ORF1b encodes an RNA-dependent RNA polymerase (RdRp) with eight conserved amino acid motifs [7, 8] that overlaps ORF2 by 8 nt [6]. A highly conserved sequence, that is suggested to be a part of the promotor sequence for sub-genomic RNA (sgRNA) synthesis, is located near the ORF2 start codon. The genome ends with 3′ UTR followed by a poly(A)-tail [6].

AstVs were first discovered in children with diarrhoea and later in various animals [9–12], including sheep [13], cattle [14], pigs [15] and roe deer [16]. The symptoms of AstV infection are usually associated with gastrointestinal tract disease, typically causing diarrhoea and vomiting, but can also proceed asymptomatically [17, 18]. However, in recent years, AstVs have been linked with diseases of the central nervous system in both humans and animals [19–25]. In avian species, AstV infection is associated with hepatitis and nephritis [18, 26].

The wide host range and genetic diversity within the *Astroviridae* family have made classification difficult. Classification into species is currently based on phylogenetic analysis of the amino acid sequence of the ORF2 genome region. AstVs are divided into two genera, *Mamastrovirus* (MAstV) and *Avastrovirus* (AAstV), infecting mammalian and avian hosts, respectively. According to the International Committee on Taxonomy of Viruses (ICTV), there are 19 different virus species divided into two genogroups within the MAstV genus and 3 virus species divided into two genogroups within the AAstV genus. However, numerous unclassified AstVs have yet to be approved as species [27].

Little research on AstVs in deer has been reported to date. In 2010, Smits et al. [16] in Denmark detected and characterized two deer AstVs (CcAstV-1 and CcAstV-2) in faecal samples of roe deer (*Capreolus capreolus*) with gastrointestinal illness. As the genetic distance between the two AstVs is similar to that between the eight human AstV serotypes, Smits et al. [16] proposed that CcAstV-1 and CcAstV-2 form two different serotypes. It is not clear whether AstV infection was responsible for causing the illness in this case [16]. The genetic similarity between bovine AstV and AstV in roe deer has been suggested as being indicative of interspecies transmission [28].

Roe deer is one of the most important game species in many European countries, including Slovenia, and is popular among hunters for its meat and antlers. As bodily excretions of infected game animals can pose a threat to the health of other animals and of humans, more attention needs to be focused towards understanding the diversity of viruses in wildlife. This could lead to early identification of new pathogens in humans and animals [29]. The complete genome and the organization of roe deer AstVs have not, so far, been described.

## Results

### RT-PCR and phylogenetic analysis of the RdRp fragment

**Fig. 1:**
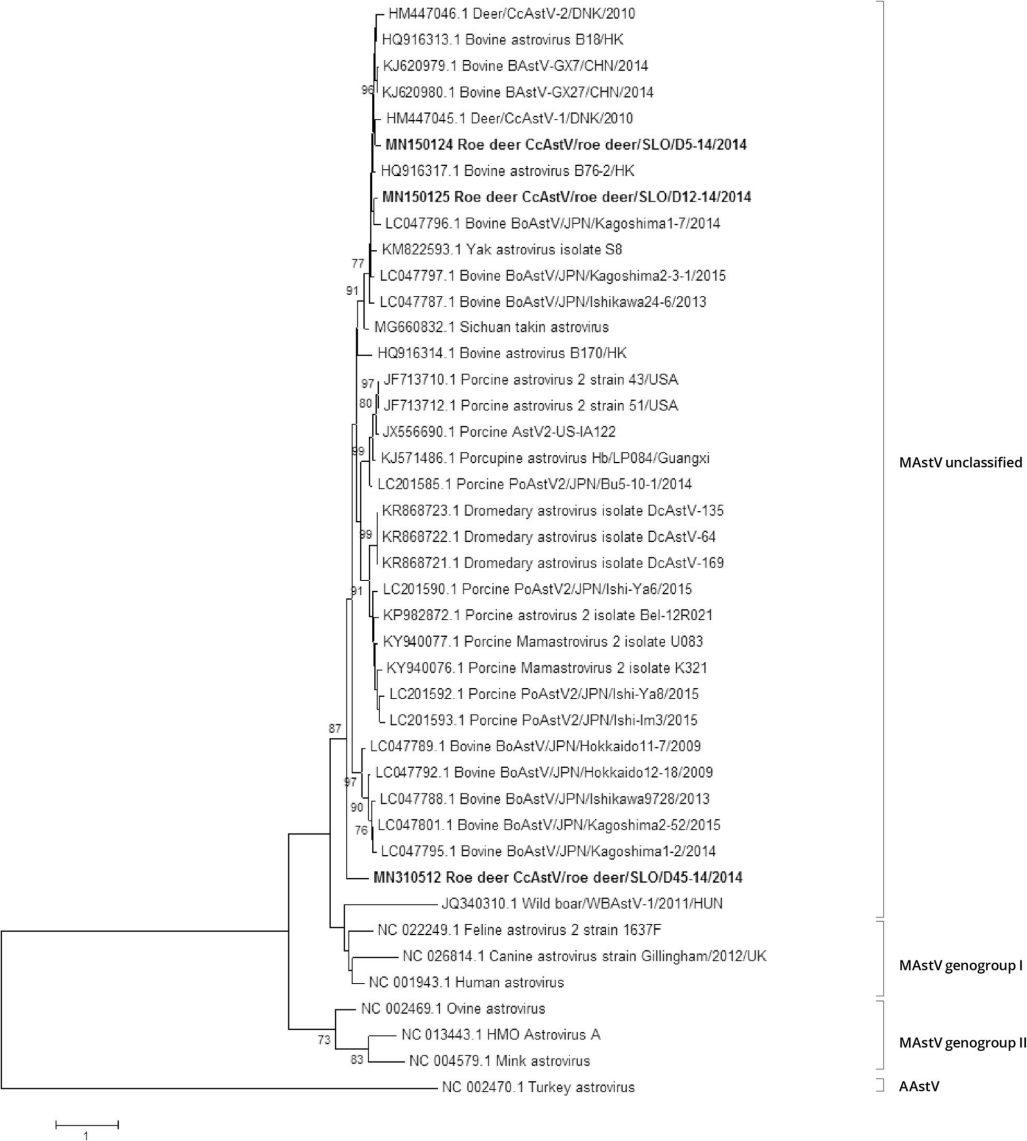
ML phylogenetic tree with the T92 + G substitution model of partial RdRp gene (328 nt) sequences. Statistical support for the phylogenetic tree was evaluated by bootstrapping, based on 1000 repetitions. Bootstrap values lower than 70 are not shown. The Slovenian roe deer AstV strains are shown in bold

A total of 111 faeces samples from roe deer, red deer, wild boar, chamois and mouflon were tested by RT-PCR analysis for the presence of AstV. No RNA was detected in samples from red deer, wild boar, chamois and mouflon, while three samples out of 65 (4.6%), namely D5–14, D12–14 and D45–14, were positive for AstV RNA. The RT-PCR products (328 nt) from the RdRp region of AstV were sequenced by the Sanger method, identifying Slovenian roe deer AstV related to that of other deer, bovine, yak, Sichuan takin, dromedary, porcine and porcupine AstV sequences, all available in GenBank (Fig. 1). Roe deer AstV SLO/D5–14 and AstV SLO/D12–14 strains share 85.1% nt identity in the partial RdRp region while their nt identity is lower than that of roe deer AstV SLO/D45–14, namely 70.5 and 71.1%, respectively. Slovenian roe deer AstV partial RdRp sequences share from 71.4 to 84.8% nt identity to those of other deer AstV and, when compared to those of bovine, yak, Sichuan takin, dromedary, porcine and porcupine AstV sequences; the nt identities range from 66.8 to 88.5%.

### AstV genome analysis

**Fig. 2:**
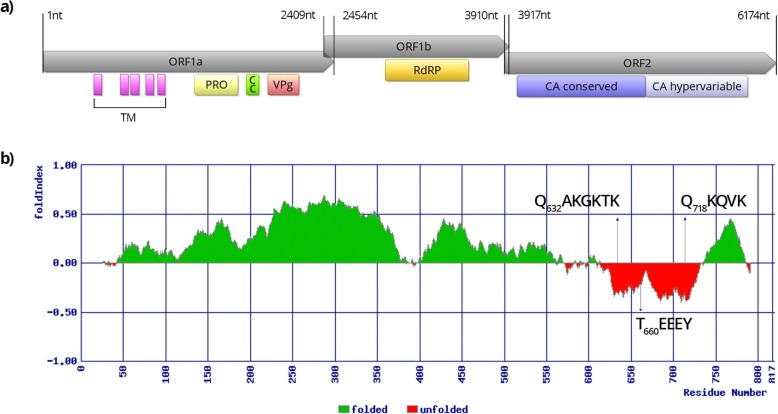
Schematic presentation of AstV SLO/D5–14 genome. (**a**) Genome organization with ORF1a, ORF1b and ORF2 with characteristic protein domains: transmembrane (TM), coiled-coil (CC), protease (PRO), viral genome-linked protein (VPg), RNA-dependent RNA polymerase (RdRp) and capsid (CA). (**b**) The possible folding of ORF1a encoded Nsp1a polyprotein with the possible N- and C- terminal cleavage sites (QAKGKTK and QKQVK) and the conserved TEEEY aa motif of unfolded VPg protein

**Fig. 3:**
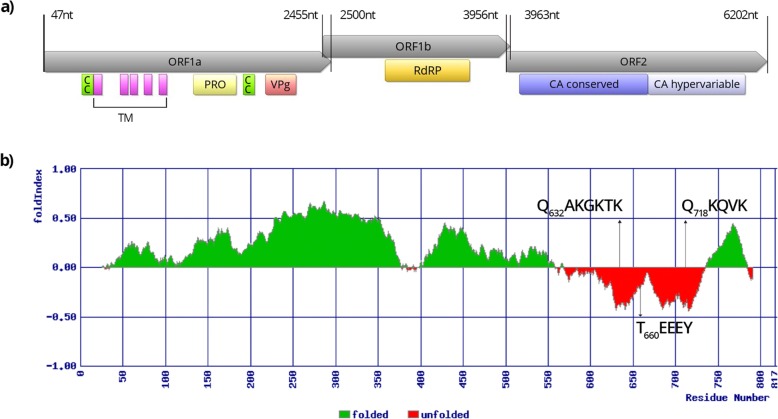
Schematic presentation of AstV SLO/D12–14 genome. (**a**) Genome organization with ORF1a, ORF1b and ORF2 with characteristic protein domains: transmembrane (TM), coiled-coil (CC), protease (PRO), viral genome-linked protein (VPg), RNA-dependent RNA polymerase (RdRp) and capsid (CA). (**b**) The possible folding of ORF1a encoded Nsp1a polyprotein with the possible N- and C- terminal cleavage sites (QAKGKTK and QKQVK) and the conserved TEEEY aa motif of unfolded VPg protein

The three samples positive for AstV RNA were subjected to whole genome sequencing with NGS, resulting in 2,510,839, 1,681,129 and 773,664 cleaned reads for samples D5–14, D12–14 and D45–14, respectively. The de novo assembled contigs were subjected to BLASTn search, which revealed two contigs, one in sample D5–14 and other in sample D12–14, representing two AstV genomes, while no AstV sequences were identified in sample D45–14. Complete coding sequences of the AstV SLO/D5–14 strain genome and the near complete genome of the AstV SLO/D12–14 strain were determined, with 19x and 118x average reads coverage, respectively. The AstV SLO/D5–14 genome is 6,245 bp long and the AstV SLO/D12–14 genome 6,274 bp long excluding the poly (A) tail. The genomes have a typical MAstV organization with ORF1a, ORF1b, followed by ORF2 from the 5′ to the 3′ end. The ORF1a and the ORF1b of both AstV genomes are 2,454 nt (818 aa) and 1,509 nt (503 aa) long, respectively and they overlap by 46 nucleotides. The ribosomal frameshift signal sequence 5′-AAAAAAC-3′, which is responsible for inducing the ribosomal frameshift during translation of the polyprotein nsp1ab, was identified near the ORF1a 3’end in both AstV genomes. In the predicted aa sequences of the non-structural polyproteins (coded by ORF1a and ORF1b) from both roe deer AstV strains, five potential transmembrane domains, a viral protease domain, coiled-coil domains (one in the AstV SLO/D5–14 and two in the AstV SLO/D12–14), a potential unfolded VPg protein and the RdRp domain were detected (Fig. 2a, Fig. 3a), whereas the NLS was not found in any of the investigated AstV genomes. In the aa sequence of the VPg of both AstV genomes, the possible N- and C- terminal cleavage sites (Q_632_AKGKTK and Q_718_KQVK) and the conserved T_660_EEEY aa motif were identified (Figs. 2, 3b). In the RdRp domain of both AstV genomes, eight conserved aa motifs [8] were detected (Table 1). The ORF2 of the AstV SLO/D5–14 genome is 2,265 nt (755 aa) long, while that of the AstV SLO/D12–14 genome is 2,247 nt (749 aa) long. The ORF1b and ORF2 sequences overlap by 8 nucleotides in both AstV genomes. The highly conserved putative AstV promoter sequence TTTGGAGNGGNGGACCANAN_4–11_ATGNC, that initiates ORF2 (where the ORF2 ATG start codon is underlined and N stands for any of the four nucleotides) is present in both roe deer AstV strains, with the following sequence: TTTGGAGGGGAGGACCAAAN_11_ATGGC and, just upstream of the ATG start codon, this sequence includes 11 nucleotides (N11) GATAAATCCTA (AstV SLO/D5–14) and GACAAGTCCTA (AstV SLO/D12–14). The AstV CA domain was identified in the predicted aa sequence of the ORF2 from both roe deer AstV strains.

**Table 1:** Conserved aa motifs of RdRp protein of AstV SLO/D5–14 and AstV SLO/D12–14

motifs	position in AstV genomes^a^	aa sequence in ORF1b^a^
I.	178	FLKKEQ
II.	197	IICPDVIYSRIGA-ALEQHQNNL-MK-KNTD
III.	230	CGWTPFFGGFAE
IV.	252	IEFDWTRFDGTIP
V.	316	GNPSGQISTTMDNNM
VI.	357	DTIVYGDDRLTS
VII.	404	IGASFCGFT
VIII.	426	KLWASLVTPC

^a^positions and aa sequences are identical in both AstV genomes

### Phylogenetic analysis of complete ORF1a, ORF1b and ORF2 genes

According to BLASTn results of the complete ORF1a, ORF1b and ORF2 sequences, the roe deer AstV strains were most closely related to deer, bovine, yak, Sichuan takin and water buffalo AstV strains, so these AstV strains, other related AstV strains and selected AstV strains of the MAstV genogroups I and II, as well as a turkey AAstV, were included in the phylogenetic analysis.

**Fig. 4:**
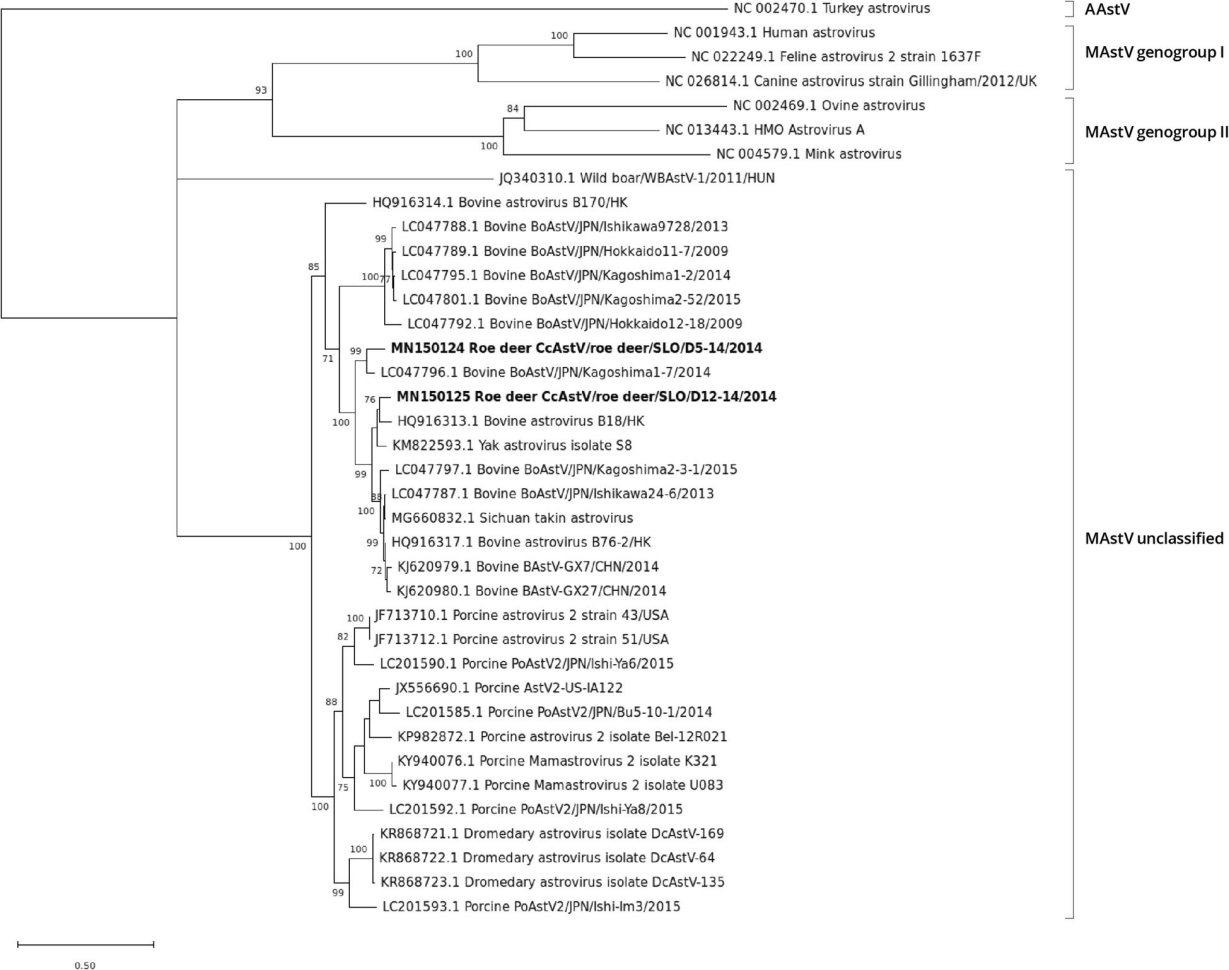
ML phylogenetic tree with the LG(+freqF) + G substitution model of ORF2 gene aa sequences. Statistical support for the phylogenetic tree was evaluated by bootstrapping, based on 1000 repetitions. Bootstrap values lower than 70 are not shown. The Slovenian roe deer AstV strains are shown in bold

The phylogenetic trees of the ORF2 gene of AstV SLO/D5–14 and AstV SLO/D12–14 strains and of other AstV strains showed that the roe deer AstV strains form a group with a high bootstrap value, with other deer AstVs, and with bovine AstVs, porcine AstV2 strains, yak AstV, Sichuan takin AstV, water buffalo AstVs, porcupine AstV and dromedary AstVs. Within this group, a cluster of closely related AstV sequences was observed. This cluster is composed of two bovine strains from Hong Kong (BAstV-B18 and BAstV-B76–2), six bovine strains (BAstGX-G1, BAstGX-J22, BAstGX-J27, BAstGX-J8, BAstGX-J7, BAstV-GX7 and BAstV-GX27) and from two water buffalo strains (BufAstGX-M552 and BufAstGX-M541) from China, one bovine strain BoAstV/JPN/Kagoshima1–7/2014 from Japan, the Sichuan takin AstV strain, two deer strains (CcAstV-1/DNK/2010 and CcAstV-2/DNK/2010) and the two roe deer AstV SLO/D5–14 and AstV SLO/D12–14 strains from this study. The roe deer AstV SLO/D5–14 strain was most closely related to deer AstV strain CcAstV-1 and the closest relative to roe deer AstV SLO/D12–14 strain was deer AstV strain CcAstV-2 with aa identities of 86.3 and 96.1%, respectively. Compared to other AstV strains within the described cluster, the aa identities of the AstV SLO/D5–14 and AstV SLO/D12–14 strains were greater than 75.4 and 74.9%, respectively. In this gene, the AstV SLO/D5–14 and AstV SLO/D12–14 strains share 77.5% aa identities (Fig. 4, Table 2).

**Table 2:** The amino acid identities of genome ORFs of two Slovenian roe deer AstV strains compared to AstV strains from GenBank

	AstV_SLO/D5–14			AstV_ SLO/D12–14		
	ORF1a [%]	ORF1b [%]	ORF2 [%]	ORF1a [%]	ORF1b [%]	ORF2 [%]
MN150124_Roe_deer_CcAstV/roe_deer/SLO/D5–14/2014				86.5	95.8	77.5
MN150125_Roe_deer_CcAstV/roe_deer/SLO/D12–14/2014	86.5	95.8	77.5			
HM447045.1_Deer/CcAstV-1/DNK/2010	NA	NA	86.3	NA	NA	74.9
HM447046.1_Deer/CcAstV-2/DNK/2010	NA	NA	77.5	NA	NA	96.1
KJ476833.1_Bovine_astrovirus_strain_BAstGX-G1	NA	NA	86.7	NA	NA	76.4
KJ476838.1_Water_buffalo_astrovirus_strain_BufAstGX-M552	NA	NA	76.9	NA	NA	75.6
KJ476837.1_Water_buffalo_astrovirus_strain_BufAstGX-M541	NA	NA	76.7	NA	NA	75.4
HQ916313.1_Bovine_astrovirus_B18/HK	84.7	95.0	75.8	92.1	95.4	75.6
LC047796.1_Bovine_BoAstV/JPN/Kagoshima1–7/2014	91.3	94.6	75.4	84.7	95.2	74.9
HQ916317.1_Bovine_astrovirus_B76–2/HK	84.1	96.0	78.2	90.5	96.4	76.9
MG660832.1_Sichuan_takin_astrovirus	83.9	93.5	85.2	90.2	93.3	76.4
KJ476835.1_Bovine_astrovirus_strain_BAstGX-J22	NA	NA	84.4	NA	NA	75.8
KJ476832.1_Bovine_astrovirus_strain_BAstGX-J27	NA	NA	84.6	NA	NA	75.8
KJ476836.1_Bovine_astrovirus_strain_BAstGX-J8	NA	NA	84.4	NA	NA	75.8
KJ476834.1_Bovine_astrovirus_strain_BAstGX-J7	NA	NA	84.8	NA	NA	76.0
KJ620980.1_Bovine_BAstV-GX27/CHN/2014	83.4	95.8	84.6	88.9	96.0	75.8
KJ620979.1_Bovine_BAstV-GX7/CHN/2014	82.8	95.4	84.8	88.9	95.6	76.0
KJ571486.1_Porcupine_astrovirus_Hb/LP084/Guangxi	NA	83.5	76.7	NA	83.1	72.6
KJ495987.1_Porcine_astrovirus_2_clone_KDC-6	NA	NA	73.0	NA	NA	72.4
LC201590.1_Porcine_PoAstV2/JPN/Ishi-Ya6/2015	73.8	82.6	72.8	74.0	81.6	73.4
JF713712.1_Porcine_astrovirus_2_strain_51/USA	73.3	84.1	71.7	73.3	83.7	69.4
KR868721.1_Dromedary_astrovirus_isolate_DcAstV-169	73.8	83.9	71.1	74.1	82.6	67.0
KR868722.1_Dromedary_astrovirus_isolate_DcAstV-64	74.3	83.7	71.3	74.8	82.4	67.0
KR868723.1_Dromedary_astrovirus_isolate_DcAstV-135	73.5	84.1	72.4	74.4	82.2	69.0
KY940076.1_Porcine_Mamastrovirus_2_isolate_K321	73.2	82.6	68.5	72.8	81.4	65.3
KY940077.1_Porcine_Mamastrovirus_2_isolate_U083	72.3	82.4	66.0	71.5	81.4	65.7
JF713710.1_Porcine_astrovirus_2_strain_43/USA	73.3	84.1	69.0	73.3	83.1	69.4
LC201585.1_Porcine_PoAstV2/JPN/Bu5–10-1/2014	72.2	84.3	69.8	73.0	83.7	70.0
JX556690.1_Porcine_AstV2-US-IA122	72.0	84.5	69.4	73.3	84.3	69.4
KM822593.1_Yak_astrovirus_isolate_S8	83.3	95.0	71.3	92.3	95.8	69.8
LC047788.1_Bovine_BoAstV/JPN/Ishikawa9728/2013	76.2	87.2	53.5	76.4	87.0	54.6
LC047789.1_Bovine_BoAstV/JPN/Hokkaido11–7/2009	75.9	88.3	53.5	75.7	87.9	55.7
LC047792.1_Bovine_BoAstV/JPN/Hokkaido12–18/2009	75.2	87.7	55.5	75.7	87.4	55.9
LC047795.1_Bovine_BoAstV/JPN/Kagoshima1–2/2014	76.0	87.7	54.2	76.0	87.0	54.8
LC047801.1_Bovine_BoAstV/JPN/Kagoshima2–52/2015	76.0	87.0	53.7	75.7	87.2	55.0
HQ916314.1_Bovine_astrovirus_B170/HK	75.7	86.4	60.4	75.6	86.4	60.4
KP982872.1_Porcine_astrovirus_2_isolate_Bel-12R021	72.2	82.6	57.6	74.1	81.8	57.0
LC201592.1_Porcine_PoAstV2/JPN/Ishi-Ya8/2015	74.0	83.1	54.8	73.2	81.8	56.5
LC201593.1_Porcine_PoAstV2/JPN/Ishi-Im3/2015	70.4	83.3	59.3	72.0	81.4	57.8
LC047787.1_Bovine_BoAstV/JPN/Ishikawa24–6/2013	83.8	95.4	47.1	90.5	95.8	45.0
LC047797.1_Bovine_BoAstV/JPN/Kagoshima2–3-1/2015	83.9	95.0	47.1	89.4	96.0	46.5
JQ340310.1_Wild_boar/WBAstV-1/2011/HUN	37.1	56.7	40.7	36.7	57.5	41.5
NC_026814.1_Canine_astrovirus_strain_Gillingham/2012/UK	28.8	59.0	35.8	29.6	59.2	36.0
NC_001943.1_Human_astrovirus	29.4	60.3	36.8	31.4	60.3	35.5
NC_022249.1_Feline_astrovirus_2_strain_1637F	28.5	58.2	36.2	29.9	59.0	34.0
NC_034975.1_California_sea_lion_astrovirus_2	NA	NA	30.8	NA	NA	30.8
NC_002469.1_Ovine_astrovirus	30.4	52.1	30.8	29.9	53.8	30.8
NC_013443.1_HMO_Astrovirus_A	29.3	50.6	28.5	30.2	50.8	29.1
NC_004579.1_Mink_astrovirus	28.6	54.8	27.4	28.8	55.2	27.8
NC_002470.1_Turkey_astrovirus	22.5	42.1	23.3	23.6	42.5	24.4

*NA* not applicable

**Fig. 5:**
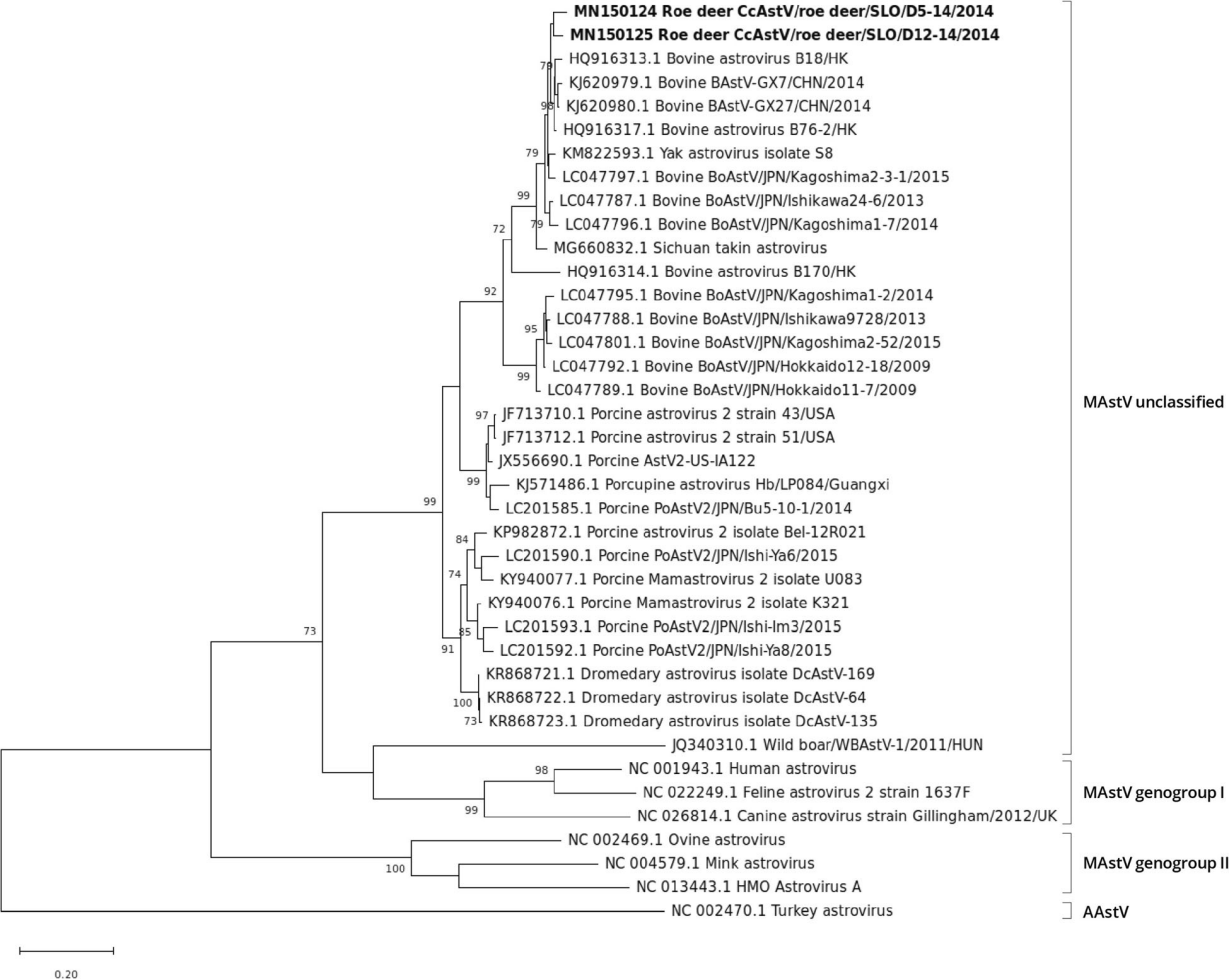
ML phylogenetic tree with the LG + G + I substitution model of ORF1a gene aa sequences. Statistical support for the phylogenetic tree was evaluated by bootstrapping, based on 1000 repetitions. Bootstrap values lower than 70 are not shown. The Slovenian roe deer AstV strains are shown in bold

**Fig. 6:**
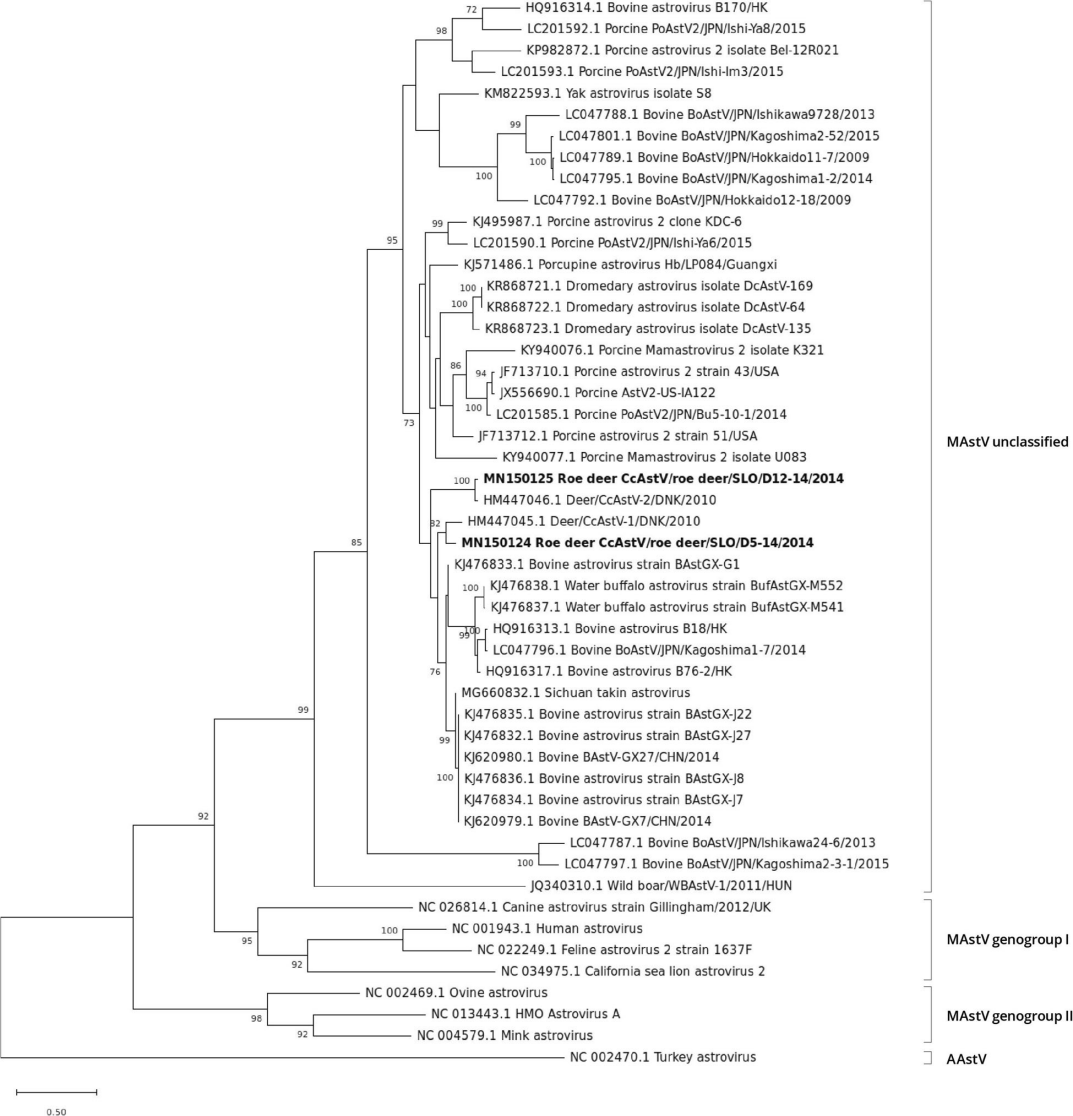
ML phylogenetic tree with the LG + G substitution model of ORF1b gene aa sequences. Statistical support for the phylogenetic tree was evaluated by bootstrapping, based on 1000 repetitions. Bootstrap values lower than 70 are not shown. The Slovenian roe deer AstV strains are shown in bold

In the ORF1a and ORF1b gene phylogenetic trees, the AstV SLO/D5–14 and AstV SLO/D12–14 strains were most closely related to the Sichuan takin AstV and to the same bovine AstV strains as those described for the ORF 2 gene phylogenetic tree, whose complete genome sequences were also determined, and, additionally, to two bovine AstV strains (BoAstV JPN/Kagoshima2–3-1/2015 and BoAstV JPN/Ishikawa24–6/2013) and to the yak AstV strain. These AstV strains form highly supported clusters of sequences with aa identities ranging from 82.8 to 92.1% for the ORF1a gene and with aa identities ranging from 93.3 to 96.4% for the ORF1b gene. The AstV SLO/D5–14 and AstV SLO/D12–14 strains shared 86.5% aa identities in the ORF1a gene and 95.8% aa identities in the ORF1b gene (Fig. 5 and Fig. 6, Table 2).

## Discussion

In this study, 65 fecal samples from roe deer were examined for the presence of AstVs with RT-PCR. The results showed a low prevalence of AstV infection among the roe deer species, as only 3 (4.6%) of the animals were positive for the presence of AstV. All of the collected samples were previously tested for the potential source of rotavirus and hepatitis E virus [30]. In samples D5–14, D12–14 and D45–14 where AstV was detected, there was no co-infection with other tested viruses.

Up to the present, there is only one description of AstV in roe deer, with only partial genome sequences determined for two CcAstV strains [16]. The complete genome and the organization of roe deer AstVs have not yet been described. The genome analysis of both roe deer AstV strains showed that they have a typical MAstV organization. The genomes of roe deer AstV from this study contain three ORFs, with ORF1a and ORF1b overlapping by 46 nt in both genomes, similarly to what was observed for the other bovine, yak and Sichuan takin AstV strains. The ORF1b and ORF2 of both roe deer AstV genomes from this study overlap by 8 nt, which is similar to what is described by Mendez et al. [6] but different from what was observed for the bovine and yak AstV strains, in which ORF1b and ORF2 overlap by 56 nt. In the genome of the Sichuan takin AstV strain this overlap was observed to be even longer. Upstream of the ORF2 in both roe deer AstV strains, a conserved sequence motif that is the putative promoter for sgRNA synthesis was predicted. This has not been described in other AstVs related to roe deer AstVs. In regard of protein domains prediction and characterization of the conserved aa motifs of the non-structural polyproteins, not many of roe deer related AstV genomes were characterized in detail. Similar as described by Tse et al. [28] for the bovine AstV, which are closely related to roe deer AstV, both of the roe deer AstV non-structural polyproteins were predicted to have the TM domains, the protease domain and the RdRp domain with characteristic aa motifs. Additionally, the putative VPg protein was predicted on the Nsp1a for both of the roe deer AstVs, whereas the NLS was not found in any of the investigated roe deer AstV genomes (Figs. 2, 3).

The phylogenetic analysis and amino acid pairwise distance analysis showed that our roe deer AstV strains are related to other deer, to bovine, water buffalo, yak, Sichuan takin, dromedary, porcine and to porcupine AstVs (Figs. 1, 4, 5 and 6).

It was proposed by Tse et al. [28] that, based on the positions in a monophyletic group and the strong branch support of bovine BAstV-B18 and BAstV-B76–2 strains and deer CCAstV strains, they should be considered, despite their different hosts, as different strains of the same virus species. To support this proposal by Tse et al. [28], at least full-length sequences of deer AstVs non-structural proteins need to be available. Later, the phylogenetic analysis of bovine and water buffalo AstV strains from China, performed by Alfred et al. [31], showed that all their isolates (of bovine and water buffalo) were closely related to those of bovine BAstV-B18 and BAstV-B76–2 strains and of deer AstVs. Their results also support the proposal that BAstV and CcAstV are different strains of the same virus with the addition of water buffalo as a possible new host of the BAstV strain of this virus species. Our results of deer AstV genome sequences constitute additional data to support this proposal of taxonomic classification. The relationship of roe deer AstVs to other AstVs was analyzed according to the criteria of the International Committee for Taxonomy of Viruses (ICTV) (http://www.ictvonline.org). In the latest ICTV proposal for the revision of MAstV taxonomy [32], both the genetic analysis of the full-length ORF2 encoding the capsid proteins and the host of origin should be considered for classification of an AstV genotype/species. According to this proposal, for the full-length ORF2, the mean aa genetic distances (p-dist) between and within different AstV genotype/species range between 37.8–75.0% (25.0–62.2% aa identity) and 0.6–31.2% (68.8–99.4% aa identity), respectively. Using more AstVs, the mean aa genetic distances between and within different AstV genotype/species were updated to range between 36.8 to 78.1% (21.9 to 63.2% aa identity) and 0–31.8% (61.9–100% aa identity), respectively [27]. According to the latter authors, the MAstV genus consists of at least 33 species, of which only 19 are currently officially recognized as genotype/species by the ICTV. The phylogenetic and aa pairwise distance analysis of ORF2 sequences revealed a cluster of closely related AstV sequences, namely the two bovine strains from Hong Kong (BAstV-B18 and BAstV-B76–2) described by Tse et al. [28], the four bovine strains (BAstGX-G1, BAstGX-J22, BAstGX-J27, BAstGX-J8 and BAstGX-J7) and two water buffalo strains (BufAstGX-M552 and BufAstGX-M541) from China, described by Alfred et al. [31], two bovine strains from China (BAstV-GX7 and BAstV-GX27), one bovine strain BoAstV/JPN/Kagoshima1–7/2014 from Japan described by Nagai et al. [33], the Sichuan takin AstV strain [34], two deer strains (CcAstV-1/DNK/2010 and CcAstV-2/DNK/2010) described by Smits et al. [16] and the two roe deer strains (SLO/D5–14 and SLO/D12–14) described in this study. The aa identities of AstV strains SLO/D5–14 and SLO/D12–14 compared to those of other AstV sequences from the described cluster were greater than 75.4 and 74.9%, respectively. Comparison with other sequences from the aforementioned cluster suggests that they belong to the same AstV genotype/species. According to Guix et al. [27], the bovine (BAstV-B18 and BAstV-B76–2), deer (CcAstV-1/DNK/2010 and CcAstV-2/DNK/2010) AstV strains belong to the MAstV 33 genotype/species. Thus, based on the close relationship of the two roe deer AstV strains (SLO/D5–14 and SLO/D12–14) to BAstV-B18, BAstV-B76–2, CcAstV-1/DNK/2010 and CcAstV-2/DNK/2010, we propose that they also belong to the MAstV 33 species/genotype. As already proposed by Smits et al. [16], that the CcAstV-1/DNK/2010 and CcAstV-2/DNK/2010 may constitute two different subtypes (serotypes), our results also suggest that roe deer AstVs belong to two subtypes based on the ORF2 phylogenetic tree clusters, namely one composed of CcAstV-1/DNK/2010 and AstV SLO/D5–14 and other composed of CcAstV-2/DNK/2010 and AstV SLO/D12–14.

When, on the phylogenetic trees of all three ORFs, clusters of the most closely related AstV strains whose complete genomes were determined were compared, the roe deer AstV SLO/D5–14 and SLO/D12–14 strains showed the same clustering as the Sichuan takin AstV, bovine AstVs BAstV-B18, BAstV-B76–2, BAstV-GX7, BAstV-GX27 and BoAstV/JPN/Kagoshima1–7/2014 strains. While, like as described previously, [33, 35], the yak AstV and two bovine AstVs from Japan (BoAstV JPN/Kagoshima2–3-1/2015 and BoAstV JPN/Ishikawa24–6/2013) showed different clustering in the ORF 1a and ORF 1b gene phylogenetic trees from that in the phylogenetic tree of the ORF 2 gene.

Based on the phylogenetic analysis, no recombination events were suspected for the roe deer AstV SLO/D5–14 and SLO/D12–14 strains, so no such analysis was performed.

The phylogenetic trees of the ORF2 genes of AstV SLO/D5–14 and AstV SLO/D12–14 strains and other AstV strains showed that the roe deer AstV strains were also related to other bovine AstVs, porcine AstV2 strains, yak AstV, porcupine AstV and dromedary AstVs forming a highly supported group of sequences distantly related to MAstV genogroups I and II, that might constitute a new MAstV genogroup, as proposed by Guan et al. [34].

In the phylogenetic tree of the partial RdRp region, the grouping of the AstV SLO/D5–14 and AstV SLO/D12–14 strains and other AstV strains was similar to that in the complete ORF1b gene phylogenetic tree. The AstV SLO/D45–14 strain did not cluster with any of the other deer AstVs, with bovine AstVs, porcine AstVs, yak AstV, porcupine AstV and dromedary AstVs strains. It could thus belong to a novel AstV genotype/species. Unfortunately, we were not able to obtain any AstV sequences with the NGS for the sample D45–14, probably due to low virus load.

## Conclusions

Based on the amino acid identities of Slovenian roe deer AstV strains and other AstV sequences from the same cluster we suggest that they belong to the same AstV genotype/species. Phylogenetic analyses in the ORF2 gene revealed that the roe deer AstV strains are also related to other bovine AstVs, porcine AstVs, yak AstV, porcupine AstV and dromedary AstVs strains, thus forming a highly supported group of currently unassigned sequences that are distantly related to MAstV genogroups I and II. This suggests the constitution of a new MAstV genogroup.

## Methods

### Sample collection

Between July 2014 and October 2015, 111 faecal samples from game animals, namely 65 roe deer (*Capreolus capreolus*), 29 wild boars (*Sus scrofa*), 10 chamois (*Rupicapra rupicapra*), 6 red deer (*Cervus elaphus*) and 1 mouflon (*Ovis musimon*) have been collected in Slovenia. Samples were collected by hunters from five hunting families in the frame of a survey in which certain game animals were screened for their potential as a source of rotavirus and hepatitis E virus [30]. In our study, these samples were investigated and tested for the presence of AstVs and, furthermore, for determination of their genome and for phylogenetic analysis. All samples were collected from animals that showed no clinical symptoms and no diarrhoea was observed. Samples D5/14, D12/14 and D45/14, were AstV was detected during the research, were collected from female animals age of under 1 year, 2 years and under 1 years old, respectively.

### RT-PCR, sanger sequencing and phylogenetic analysis of the RdRp fragment

10% suspensions of faecal samples were prepared with RPMI medium 1640 (Thermo Fisher Scientific, Carlsbad, CA, USA). The suspensions were homogenized and centrifuged at 2000×g for 10 min and the supernatant stored at − 70 °C. The latter was used for nucleic acid extraction using the QIAamp viral RNA mini kit according to the manufacturer’s instructions (Qiagen, Germany). An AstV specific RT-PCR amplifying a part of the RdRp region of the AstV genome was used for detecting AstV [36], using specific primers (SF0073: 5′-GAT TGG ACT CGA TTT GAT GG-3′, SF0076: 5′- CTG GCT TAA CCC ACA TTC C-3′). After each PCR product was electrophoresed in a 1.8% agarose gel, the RT-PCR products judged to be positive by the expected size of the DNA fragment (409 bp) were purified and sequenced by the Sanger method (Macrogen, Netherlands). The nucleotide sequences thus obtained were analysed using Seqman and EditSeq implemented in the DNASTAR program (Lasergene, WI, USA) and compared with the sequences published in the GenBank (NCBI). Multiple alignments were created using MEGA v.7.0.21 [37]. The best fitting nucleotide substitution model was determined based on the lowest BIC scores. Phylogenetic trees were constructed with MEGA v.7.0.21 [37], using the ML method with the Tamura 3 (T92) substitution model with the gamma parameter. Statistical support for the phylogenetic tree was evaluated by bootstrapping based on 1000 repetitions.

The sequence was deposited in GenBank under accession number MN310512.

### Next generation sequencing

For complete genome sequencing with next generation sequencing (NGS), total RNA was extracted from AstV positive samples D5/14, D12/14 and D45/14 with TRIzol™ Reagent (Invitrogen, Carlsbad, USA), according to the manufacturer’s instructions. The cDNA Synthesis System (Roche, Manheim, Germany) and Random-Hexamer-Primer (Roche, Manheim, Germany) were used for cDNA synthesis, according to the manufacturer’s instructions. Covaris M220 focused-ultrasonicator (Covaris, USA) was used to fragment the cDNA, targeting peak fragment lengths of 400 bp. Fragmented cDNA was purified and concentrated with magnetic beads Agencourt AMPure XP Beads (Beckman Coulter, MA, USA). The GeneRead™ DNA Library L Prep Kit (Qiagen, Hilden, Germany) was used for barcoded NGS library preparation, according to the manufacturer’s instructions. Agencourt AMPure XP Beads (Beckman Coulter, MA, USA) were used for purification and double size selection of the NGS library fragments. NGS library concentration was determined with the QIAseq Library Quant Assay Kit (Qiagen, Hilden, Germany), using the Qubit v.3.0 fluorometer (Thermo Fisher Scientific, CA, USA). Emulsion PCR and enrichment were carried out using the Ion PGM™ Hi-Q™ View OT2 Kit reagents (ThermoFisher Scientific – Ion Torrent, CA, USA) according to the manufacturer’s instructions. The NGS library was sequenced on the Ion PGM platform using the Ion PGM™ Hi-Q™ View Sequencing Kit reagents (ThermoFisher Scientific – Ion Torrent, CA, USA).

### Bioinformatic analysis of NGS data and of assembled genomes

Sequenced reads were quality checked and trimmed using the Ion Torrent Suite v.5.6.0. Additionally, low quality bases were trimmed and duplicate reads removed with Geneious software suite v.11.0.5 (Biomatters Ltd., New Zealand). SPAdes software v.3.10.0 was used for de novo assembly of the reads. The assembled contigs were subjected to BLASTn search to determine those that represent the AstV sequences. Finally, to eliminate assembly errors, all sequenced reads were mapped against the assembled genomes with the Geneious reference mapper (Geneious software suite v.11.0.5, Biomatters Ltd., Auckland, New Zealand).

Geneious software suite v.11.0.5 (Biomatters Ltd., Auckland, New Zealand) was used for further downstream bioinformatic analyses of the assembled AstV genomes. ORFs were predicted with the Geneious ORF finder and protein family analysis performed with InterProScan [38]. The nt and aa alignments were manually inspected for identification of the conserved aa motifs and of the conserved nt sequences. cNLS Mapper (http://nls-mapper.iab.keio.ac.jp/cgi-bin/NLS_Mapper_form.cgi) [39] was used to predict the NLS. The folding of the ORF1a encoded Nsp1a polyprotein and the potential cleavage sites of unfolded VPg protein were predicted with FoldIndex (https://fold.weizmann.ac.il/fldbin/findex) [40] and ExPASy PeptideCutter (https://web.expasy.org/peptide_cutter/) [41], respectively.

Sequences were deposited in GenBank under accession numbers MN150124 and MN150125.

### Phylogenetic analysis of complete ORF1a, ORF1b and ORF2 genes

Nucleotide sequences of selected AstV were retrieved from GenBank according to BLASTn search that identified those relevant for further analyses. Amino acid (aa) sequence alignment of the complete ORF1a, ORF1b and ORF2 genes were constructed with the MUSCLE program [42]. Based on the alignments, the aa genetic distances were calculated using the *p*-distance model implemented in MEGA v.7.0.21 [37]. Phylogenetic analyses of the ORF1a, ORF1b and ORF2 genes were performed with MEGA v.7.0.21 [37]. The best fitting aa substitution model, based on the lowest BIC scores, was determined. Phylogenetic trees of the AstV ORF1a, ORF1b and ORF2 aa sequences were constructed using the ML method with the LG + G + I, LG + G and LG(+freqF) + G substitution model, respectively. Branch statistics were calculated by bootstrap analysis of 1000 replicates.

## Data Availability

All data generated or analyzed during this study are included in this published article. All the materials are stored in the Institute of Food safety, feed and environment, Veterinary Faculty, University of Ljubljana, Slovenia. Nucleotide sequences are deposited in GenBank and available under accession numbers MN310512, MN150124 and MN150125.

## Abbreviations

AAstV: Avastrovirus
AstV: Astrovirus
CA: Capsid
EM: Electron microscop
ICTV: International Committee on Taxonomy of Viruses
MAstV: Mamastrovirus
NGS: Next generation sequencing technology
nt: Nucleotids
ORF: Open reading frames
RdRp: RNA-dependent RNA polymerase
sgRNA: Sub-genomic RNA
UTR: Untranslated region

## References

[1] Appleton H, Higgins PG (1975). Letter: viruses and gastroenteritis in infants. Lancet..

[2] Willcocks MM, Ashton N, Kurtz JB, Cubitt WD, Carter MJ (1994). Cell culture adaptation of astrovirus involves a deletion. J Virol.

[3] Monroe SS, Jiang B, Stine SE, Koopmans M, Glass RI (1993). Subgenomic RNA sequence of human astrovirus supports classification of Astroviridae as a new family of RNA viruses. J Virol.

[4] Willcocks MM, Carter MJ (1993). Identification and sequence determination of the capsid protein gene of human astrovirus serotype 1. FEMS Microbiol Lett.

[5] Méndez E, Aguirre-Crespo G, Zavala G, Arias CF (2007). Association of the astrovirus structural protein VP90 with membranes plays a role in virus morphogenesis. J Virol.

[6] Méndez E, Murillo A, Velázquez R, Burnham A, Arias CF, Schultz-Cherry S (2012). Replication cycle of Astroviruses. Astrovirus research.

[7] Koonin EV (1991). The phylogeny of RNA-dependent RNA polymerases of positive-strand RNA viruses. J Gen Virol..

[8] Jiang B, Monroe SS, Koonin EV, Stine SE, Glass RI (1993). RNA sequence of astrovirus: distinctive genomic organization and a putative retrovirus-like ribosomal frameshifting signal that directs the viral replicase synthesis. Proc Natl Acad Sci U S A.

[9] Kurtz JB, Lee TW (1987). Astroviruses: human and animal. CIBA Found Symp.

[10] Toffan A, Jonassen CM, De Battisti C, Schiavon E, Kofstad T, Capua I (2009). Genetic characterization of a new astrovirus detected in dogs suffering from diarrhoea. Vet Microbiol.

[11] Hoshino Y, Zimmer JF, Moise NS, Scott FW (1981). Detection of astroviruses in feces of a cat with diarrhea. Arch Virol.

[12] Bosch A, Pintó RM, Guix S (2014). Human astroviruses. Clin Microbiol Rev.

[13] Snodgrass DR, Gray EW (1977). Detection and transmission of 30 nm virus particles (astroviruses) in faeces of lambs with diarrhoea. Arch Virol.

[14] Woode GN, Bridger JC (1978). Isolation of small viruses resembling astroviruses and caliciviruses from acute enteritis of calves. J Med Microbiol.

[15] Bridger JC (1980). Detection by electron microscopy of caliciviruses, astroviruses and rotavirus-like particles in the faeces of piglets with diarrhoea. Vet Rec.

[16] Smits SL, van Leeuwen M, Kuiken T, Hammer AS, Simon JH, Osterhaus AD (2010). Identification and characterization of deer astroviruses. J Gen Virol..

[17] Walter JE, Mitchell DK (2003). Astrovirus infection in children. Curr Opin Infect Dis.

[18] Moser LA, Schultz-Cherry S (2005). Pathogenesis of astrovirus infection. Viral Immunol.

[19] Quan PL, Wagner TA, Briese T, Torgerson TR, Hornig M, Tashmukhamedova A (2010). Astrovirus encephalitis in boy with X-linked agammaglobulinemia. Emerg Infect Dis.

[20] Wunderli W, Meerbach A, Güngör T, Guengoer T, Berger C, Greiner O (2011). Astrovirus infection in hospitalized infants with severe combined immunodeficiency after allogeneic hematopoietic stem cell transplantation. PLoS One.

[21] Naccache SN, Peggs KS, Mattes FM, Phadke R, Garson JA, Grant P (2015). Diagnosis of neuroinvasive astrovirus infection in an immunocompromised adult with encephalitis by unbiased next-generation sequencing. Clin Infect Dis.

[22] Blomström AL, Widén F, Hammer AS, Belák S, Berg M (2010). Detection of a novel astrovirus in brain tissue of mink suffering from shaking mink syndrome by use of viral metagenomics. J Clin Microbiol.

[23] Pfaff F, Schlottau K, Scholes S, Courtenay A, Hoffmann B, Höper D (2017). A novel astrovirus associated with encephalitis and ganglionitis in domestic sheep. Transbound Emerg Dis.

[24] Li L, Diab S, McGraw S, Barr B, Traslavina R, Higgins R (2013). Divergent astrovirus associated with neurologic disease in cattle. Emerg Infect Dis.

[25] Arruda B, Arruda P, Hensch M, Chen Q, Zheng Y, Yang C (2017). Porcine Astrovirus type 3 in central nervous system of swine with Polioencephalomyelitis. Emerg Infect Dis.

[26] De Benedictis P, Schultz-Cherry S, Burnham A, Cattoli G (2011). Astrovirus infections in humans and animals - molecular biology, genetic diversity, and interspecies transmissions. Infect Genet Evol.

[27] Guix S, Bosch A, Pinto RM, Schultz-Cherry S (2013). Astrovirus Taxonomy. Astrovirus research.

[28] Tse H, Chan WM, Tsoi HW, Fan RY, Lau CC, Lau SK (2011). Rediscovery and genomic characterization of bovine astroviruses. J Gen Virol..

[29] Kuiken T, Leighton FA, Fouchier RA, LeDuc JW, Peiris JS, Schudel A (2005). Public health. Pathogen surveillance in animals. Sci..

[30] Sturm S (2016). Detection of rotavirus and hepatitis E virus in game animals.

[31] Alfred N, Liu H, Li ML, Hong SF, Tang HB, Wei ZZ (2015). Molecular epidemiology and phylogenetic analysis of diverse bovine astroviruses associated with diarrhea in cattle and water buffalo calves in China. J Vet Med Sci.

[32] Bosch A, Guix S, Krishna NK, Méndez E, Monroe SS, Pantin-Jackwood M (2010). Nineteen new species in the genus Mamastrovirus in the Astroviridae family, ICTV 2010.

[33] Nagai M, Omatsu T, Aoki H, Otomaru K, Uto T, Koizumi M (2015). Full genome analysis of bovine astrovirus from fecal samples of cattle in Japan: identification of possible interspecies transmission of bovine astrovirus. Arch Virol.

[34] Guan TP, Teng JLL, Yeong KY, You ZQ, Liu H, Wong SSY (2018). Metagenomic analysis of Sichuan takin fecal sample viromes reveals novel enterovirus and astrovirus. Virol..

[35] Chen X, Zhang B, Yue H, Wang Y, Zhou F, Zhang Q (2015). A novel astrovirus species in the gut of yaks with diarrhoea in the Qinghai–Tibetan plateau, 2013. J Gen Virol.

[36] Finkbeiner SR, Le BM, Holtz LR, Storch GA, Wang D (2009). Detection of newly described astrovirus MLB1 in stool samples from children. Emerg Infect Dis.

[37] Kumar S, Stecher G, Tamura K (2016). MEGA7: molecular evolutionary genetics analysis version 7.0 for bigger datasets. Mol Biol Evol.

[38] Jones P, Binns D, Chang HY, Fraser M, Li W, McAnulla C (2014). InterProScan 5: genome-scale protein function classification. Bioinformatics..

[39] Kosugi S, Hasebe M, Tomita M, Yanagawa H (2009). Systematic identification of cell cycle-dependent yeast nucleocytoplasmic shuttling proteins by prediction of composite motifs. Proc Natl Acad Sci U S A.

[40] Prilusky J, Felder CE, Zeev-Ben-Mordehai T, Rydberg EH, Man O, Beckmann JS (2005). FoldIndex: a simple tool to predict whether a given protein sequence is intrinsically unfolded. Bioinformatics..

[41] Gasteiger E, Hoogland C, Gattiker A, Duvaud S, Wilkins MR, Appel RD, Walker JM (2005). Protein Identification and Analysis Tools on the ExPASy Server. The Proteomics Protocols Handbook.

[42] Edgar RC (2004). MUSCLE: multiple sequence alignment with high accuracy and high throughput. Nucleic Acids Res.

